# IMPROVING DEMENTIA DETECTION IN PRIMARY CARE: PROCESS EVALUATION FOR THE ERADAR BRAIN HEALTH TRIAL

**DOI:** 10.1093/geroni/igad104.1270

**Published:** 2023-12-21

**Authors:** Deborah Barnes, Mikael Anne Greenwood-Hickman, Clarissa Hsu, Leah Karliner, Deborah King, Sei Lee, Rebecca Coley, Sascha Dublin

**Affiliations:** University of California, San Francisco, San Francisco, California, United States; Kaiser Permanente Washington Health Research Institute, Seattle, Washington, United States; Kaiser Permanent Washington Health Research Institute, Seattle, Washington, United States; University of California San Francisco, San Francisco, California, United States; Kaiser Permanente Washington, Seattle, Washington, United States; University of California, San Francisco, San Francisco, California, United States; Kaiser Permanente Washington Health Research Institute, Seattle, Washington, United States; Kaiser Permanente Washington, Seattle, Washington, United States

## Abstract

The eRADAR Brain Health Study is an ongoing embedded, pragmatic trial testing whether targeted screening improves detection and diagnosis of dementia in primary care clinics at Kaiser Permanente Washington and the University of California, San Francisco. The validated eRADAR algorithm, which uses electronic health record data to calculate a risk score for undiagnosed dementia, identifies high-risk patients age ≥65. Participants are randomized at the primary care physician (PCP) level to receive usual care or an invitation to a brain health visit for functional and cognitive assessment. We assessed patient acceptance (% completed brain health visit) and satisfaction (responses to post-visit survey) in patients enrolled to date. Of 575 high-risk patients invited, 157 (27%) agreed and 116 (20%) completed a brain health visit. The primary reason for declining the visit was lack of interest. Those who completed the visit had a mean (SD) age of 83.4 (5.1) years; 70 (60.34%) were women; 82 (70.7%) attended with a care partner. Seventeen (15%) had results consistent with dementia and were referred to their PCP for further evaluation. Fifty-five (47%) had results consistent with mild cognitive impairment (MCI) and were counseled to follow up with their PCP in 1 year. Analysis of the first 40 survey responses indicated high satisfaction: 33 (83%) agreed the visit was helpful and 36 (90%) did not feel upset/concerned by the visit. This suggests that, although acceptance was low, those who participated had high rates of possible dementia or MCI and reported high satisfaction with the targeted screening process.

